# Media Representations of Aging and Their Psychological Impact: Age Anxiety Among Older Korean Adults

**DOI:** 10.3390/bs15070932

**Published:** 2025-07-10

**Authors:** Soondool Chung, Miri Kim, Yuri Jang, Nan Sook Park, Hyunwoo Yoon

**Affiliations:** 1 Department of Social Welfare, Ewha Womans University, Seoul 03760, Republic of Korea; sdchung@ewha.ac.kr (S.C.); yurij@usc.edu (Y.J.); 2 Department of Social Welfare, Korea National University of Transportation, Chungbuk 27469, Republic of Korea; 3 Edward R. Roybal Institute on Aging, Suzanne Dworak-Peck School of Social Work, University of Southern California, Los Angeles, CA 90089, USA; 4 School of Social Work, University of South Florida, Tampa, FL 33620, USA; nanpark@usf.edu; 5 Department of Social Welfare, Kongju National University, Gongju-si 32588, Republic of Korea

**Keywords:** media, older people, aging anxiety, age stereotype, mental health, Asia

## Abstract

This study investigates the association between media representation, perceived age stereotypes, and aging anxiety among Korean older adults. A total of 600 older adults aged 65 years and older were analyzed via structural equation modelling and the bootstrapping method using a cross-sectional, secondary dataset. Regarding the direct effects, media representation was positively associated with perceived age stereotypes, aging anxiety related to financial matters, and aging anxiety in relation to psychosocial factors. In addition, perceived age stereotypes were positively associated with aging anxiety in regard to psychosocial factors. In terms of indirect effects, perceived age stereotypes only mediated the relationship between media representation and aging-anxiety-related psychosocial factors. This study’s findings are significant for alleviating aging anxiety in an aging society, offering practical strategies for mitigating such concerns.

## 1. Introduction

### 1.1. Background and Significance

Aging anxiety, defined as “the combined concern and anticipation of losses centered around the aging process,” (Lasher & Faulkender, 1993, p. 247) or “an anxious state created through worry about anticipated threats associated with the process of aging” (Watkins et al., 1998, p. 320), is a psychological factor that plays a significant role in one’s ability to understand and adapt to the aging process (Bergman & Bodner, 2022). This phenomenon can have negative impacts on the psychological well-being of individuals and influence the attitudes and behaviors of older adults (Yawar et al., 2022). Previous studies on aging anxiety have primarily focused on younger and middle-aged adults’ fears and concerns regarding their prospects of aging, and the perspectives of older adults have been studied less thoroughly (Bergman & Bodner, 2022; Bergman & Segel-Karpas, 2021). However, examining aging anxiety in the older population is not only relevant but also urgently required to better understand the aging process of older adults, who face longer later-life periods than ever before.

In particular, aging anxiety among older adults should be studied in depth in the context of South Korea, which is projected to become the nation with the longest-living population in the world in 50 years (Statistics Korea, 2021). Furthermore, as of 2021, South Korea had a life expectancy of 83.6 years, one of the highest figures among Organisation for Economic Co-operation and Development (OECD) countries (Statistics Korea, 2022), suggesting more Korean older adults will face more time after retirement in the future. Therefore, studying aging anxiety among older adults can provide meaningful insights into the emerging problems of an aging society by revealing the diverse aspects of concerns in later life and the associated factors, helping to prepare for an extended later life.

### 1.2. Theoretical Framework

The current study recognizes the impact of COVID-19 on media use among older adults and investigates the relationship between the representation of older adults in media, perceived age stereotypes, and aging anxiety. The effects of media on individuals and society have become more prominent in the era of COVID-19. Online media consumption increased at an unprecedented rate as social and physical distancing measures were widely implemented (Neill et al., 2021). Older adults were particularly affected by public safety measures due to their physical vulnerability, and more of them were motivated to start utilizing various kinds of media to stay connected with family and friends (Cotten et al., 2022). Often times, media serves as a resource for social networking and cognitive stimulation (Quinn, 2018); however, it can also be associated with psychological distress and adverse mental health outcomes, such as depression and anxiety (Allen et al., 2019).

Stereotype embodiment theory (SET) is often applied to support the links between messages conveyed in the media, playing an influential role in impacting the mental health of users. According to SET, continuous exposure to societal resources that contain age-stereotypical information, such as media, contributes to the development of age-related stereotypes that are internalized and have lasting effects on the health of older populations (Levy, 2009; Levy et al., 2022; Shimizu et al., 2022). The media frequently portrays aging as an undesirable process, while youth is considered to be a desirable state (Fraser et al., 2016). Although the typically portrayed characteristics of older people, including frailty, illness, vulnerability, and dependence, are biased representations that oversimplify old age and do not fully reflect the diversities within the older population, continued exposure to such information can lead individuals to believe that these representations are the norms of society. Levy (2009) emphasizes that age-related stereotypes are absorbed across one’s life span, often without conscious awareness, and become especially powerful as individuals begin to identify themselves as part of the aging population. Over time, these internalized beliefs not only shape how people perceive others but also influence how they view their own aging, influencing psychological well-being, health behaviors, and even physical outcomes (Bergman & Segel-Karpas, 2021; Levy, 2009). For instance, studies show that individuals who hold negative age stereotypes, or internalize negative views about aging, are more likely to experience poorer mental well-being and lower cognitive and physical performance as they age, including more intense feelings of anxiety, loneliness, and distress as well as a significant increase in blood pressure and a decrease in memory performance (Shimizu et al., 2022; Levy et al., 2000).

Since the end of the COVID-19 pandemic, more studies have begun to investigate the relationship between media, age stereotypes, and mental health. The study by Levy and colleagues (Levy et al., 2022) is similar to the current study in that it explored the association between media-based age stereotypes and mental health among younger, middle-aged, and older participants. However, the study by Levy et al. (2022) was a one-time experimental intervention using pre-selected positive, neutral, and negative media content. In contrast, the present study investigates older adults’ subjective perceptions of repeated exposure to negative portrayals of aging in everyday media environments. Instead of measuring short-term reactions to experimental stimuli, this study examines self-reported media exposure in everyday life, providing insight into its possible links to psychological well-being. Specifically, the aim is to understand how such exposure affects the way older adults perceive age stereotypes of themselves, which in turn may lead to higher levels of aging anxiety, as suggested by SET.

### 1.3. Study Objectives and Hypotheses

In this study, we aim to address key gaps in the literature by investigating the psychological mechanisms through which media portrayals of aging influence aging anxiety among older adults, using SET as the guiding theoretical framework. Specifically, this study explores whether exposure to negative media representations of aging is associated with internalized age stereotypes, which in turn heighten anxiety about one’s own aging process. By focusing on everyday media exposure rather than short-term experimental stimuli, this study provides insights into how cumulative and subjective perceptions of media representations may have associations with psychological outcomes in later life.

Furthermore, this study expands prior research by examining two distinct dimensions of aging anxiety: psychosocial and economic. Most existing studies primarily focused on psychosocial aging anxiety, and relatively few have examined aging anxiety stemming from financial concerns. This conceptual distinction is particularly relevant in the Korean context, where both types of anxiety may be intensified due to rapid demographic shifts and weak social safety nets.

Jung (2020) emphasized that Korean older adults often experience financial and psychological threats simultaneously and highlighted the need to examine multiple domains of aging anxiety rather than treating it as a single construct. Building on this, the present study introduces financial aging anxiety as an independent yet related aspect of overall aging anxiety. Given Korea’s exceptionally high poverty rate among older adults (43.4%) and widespread retirement insecurity (OECD, 2021), understanding the distinct pathways leading to psychosocial and financial aging anxiety will provide crucial information for developing more effective policies and interventions.

The hypotheses of this study are as follows.

**H1.**  *Greater exposure to negative representations of aging in the media is associated with higher levels of perceived age stereotypes.*

**H2.**  *Greater exposure to negative representations of aging in the media is associated with greater financial aging anxiety.*

**H3.**  *Greater exposure to negative representations of aging in the media is associated with higher levels of psychosocial aging anxiety.*

**H4.**  *Having higher levels of perceived age stereotypes is associated with greater financial aging anxiety.*

**H5.**  *Having higher levels of perceived age stereotypes is associated with greater psychosocial aging anxiety.*

**H6.**  *Perceived age stereotypes mediate the relationship between media representation and financial aging anxiety.*

**H7.**  *Perceived age stereotypes mediate the relationship between media representation and psychosocial aging anxiety.*

The results of this study will provide a deeper understanding of the role of media representations in generating aging anxiety and perceived age stereotypes among older adults while also illuminating the interplay between media representation, perceived age stereotypes, and aging anxiety. The findings of this study, obtained through an empirical analysis based on SET, can contribute to promoting well-being in later life.

## 2. Methods

### 2.1. Data and Sample

In this study, we utilized a cross-sectional, secondary dataset obtained from the 2020 Age Integration and Intergenerational Solidarity Survey, including a sample of 600 older adults aged over 65 years. The survey was conducted by the OO Research Institute in South Korea and approved by the OO University Institutional Review Board (No. 202005-0003-01). Informed consent was obtained from the participants appropriately. Data collection took place between 17 June 2020 and 31 July 2020, using a multistage quota sampling technique that considered factors relating to age, region, and gender. All survey instruments were administered in Korean. Control variables included age, region (1 = rural city, 2 = medium-sized city, and 3 = metropolitan city), gender (0 = female and 1 = male), self-rated physical health status (1 = poor, 2 = fair, 3 = good, 4 = very good, and 5 = excellent), and occupational status (0 = not employed and 1 = employed). Face-to-face interviews were conducted by pre-trained researchers to ensure that all questionnaires were clearly understood by the study participants and confirm that no questions were unanswered at the site of the interview. Trained interviewers confirmed on site that all questions were completed and that no responses were omitted during data collection. Consequently, the dataset was complete, with no missing data.

### 2.2. Measures

#### 2.2.1. Aging Anxiety

Jung’s (2020) aging anxiety scale, which was developed and validated for use in the Korean context, was used to assess aging anxiety with respect to financial and psychosocial factors. To investigate the specific roles of each of these two factors, the sub-scales were analyzed separately.

The financial-related sub-scale consisted of seven items that assessed anxiety related to financial insecurity in later life. These items included concerns about reduced income, changes in lifestyle, unemployment, increased medical expenses, and possible caregiving burdens for parents. Sample items include, “I feel anxious because I am not financially prepared for my later life,” “I am afraid that I will not be able to enjoy the same hobbies and leisure activities as I do now in my later years due to financial hardship,” “I am afraid I will face financial difficulties in paying for medical expenses in my later years,” and “I am afraid I will not be able to get a job even if I wanted to work in my later years.” The sub-scale was adopted from the economic insecurity scale developed by Hwang (1995) and the economic well-being scale developed by Yu (2002).

The psychosocial-related sub-scale consisted of ten items that assessed anxiety related to loss of close relationships, decreased social connections, health issues, and displacement from one’s current place of residence. Sample items included, “I am afraid of losing my spouse or close family members (parents, children, siblings, etc.) in my later years,” “I am afraid that my involvement in social activities will decrease or I will be faced with less opportunities for social recognition in my later years,” “I am afraid of suffering from disease in my later years,” and “I am afraid of being alienated from friends, neighbours, and local community in my later years.” The sub-scale was adopted from the anxiety about aging scale (AAS) developed by Lasher and Faulkender (1993), translated by W. Kim (2011) for use in the Korean context.

The scale was measured using a five-point Likert scale from 1 (hardly ever) to 5 (very frequently). Higher scores indicated higher levels of aging anxiety among respondents. The Cronbach alpha values for this study were 0.849 and 0.862 for aging anxiety related to financial matters and psychosocial factors, respectively.

#### 2.2.2. Media Representation

The media age discrimination scale developed by Lee et al. (2020) was used to measure how older adults and aging are represented in the media. The original scale created by Lee et al. (2020) only considered television in the questionnaire; however, the scale used in this study was expanded to include the platforms of news, social media services, and mobile messengers due to the significant changes in media platforms over the past decade. Sample items include, “Older people are not reflected in TV programs,” “There are not many TV programs for older adults,” “TV programs do not say good things about older people,” “Older people that appear on TV are not attractive,” “Older adults in the news are not portrayed positively,” “There is little discussion about older people on social networking services,” and “Older adults mentioned in mobile messenger conversations are not portrayed as attractive.” All participants were users of all four platforms, and there were no missing responses. A total of 16 items are included in this scale, and the scale was measured using a five-point Likert scale ranging from 1 (not at all) to 5 (extremely). Higher scores indicated more negative representations and perceived discrimination of older adults portrayed in media. The Cronbach alpha of the overall scale, consisting of 16 items, was 0.898. When analyzed by media type, the Cronbach’s alpha coefficients were 0.720 for television, 0.727 for news, 0.776 for social media, and 0.795 for mobile messengers.

#### 2.2.3. Perceived Age Stereotypes

The age stereotype scale created by An et al. (2018) was used to measure the negative attributes associated with Korean older adults. The scale consists of 15 items, including five dimensions of age stereotypes in the areas of ability, personality, appearance, authoritarian dependency, and obsession with one’s children. Sample items include, “Older people do not listen to other people well,” “Older people are fragile and weak,” “Older people have slouched postures,” “Older people try to depend on their children,” and “Older people only care about the success of their children.” The scale was measured using a five-point Likert scale ranging from 1 (not at all) to 5 (extremely). Higher scores indicate that a participant perceives themself in a manner more in line with age stereotypes. The Cronbach alpha for this study was 0.833.

### 2.3. Analytical Approach

To examine the associations between media representation, perceived age stereotypes, and aging anxiety, structural equation modelling (SEM) was applied, adopting the two-step modelling approach developed by Anderson and Gerbing (1988). SPSS 18.0 and Mplus 7.0. software packages were used to obtain descriptive statistics and perform the SEM process. Firstly, items categorized as latent variables were aggregated to generate three parcels per construct using the balancing approach (Little et al., 2013). Secondly, the measurement model was tested using confirmatory factor analysis (CFA). Thirdly, the structural model was tested, and a bootstrapping method with 5000 iterations was employed to test indirect effects. In evaluating model fit, model fit indices of CFI ≥ 0.95, SRMR ≤ 0.0831, and RMSEA ≤ 0.0832 were considered to be a good fit, as researchers often disregard non-significant values of $\chi^{2}$ because of its sensitivity to large samples (Hu & Bentler, 1999; Browne & Cudeck, 1993). Indirect effects were considered to be significant when the 95% confidence interval did not contain 0.

## 3. Results

### 3.1. Descriptive Information

The sociodemographic characteristics of the participants are presented in Table 1. The mean age of the participants was 73.77 years (SD = 5.797), with a range of 65 to 84. A total of 44.3% of the participants were male, and 55.7% were female, and 62.3% of participants responded that they had a spouse, while 37.7% said they did not. The majority of the participants (76.8%) reported that they owned their own homes, with only 23.2% reporting that they did not own their own homes. In terms of occupational status, 59.5% of the participants were not employed, while 40.5% were still employed. The participants reported an average subjective health status of 3.09 (SD = 0.908) on a scale from 1 to 5 and an average subjective economic status of 2.40 (SD = 0.681) on a scale from 1 to 5.

### 3.2. Measurement Model

The results of the measurement model tested using CFA are presented in Table 2. All factor loadings were significant at the 0.001 level. The model fit indices of $\chi^{2}\left(48\right)$ = 126.361 (*p* < 0.001), CFI = 0.982, RMSEA = 0.052, and SRMR = 0.026 indicate a good fit for the model across all indices. Therefore, the measurement model was considered to have a suitable representation of the latent constructs of media representation, perceived age stereotypes, and aging anxiety.

### 3.3. Structural Model

**Figure 1:**
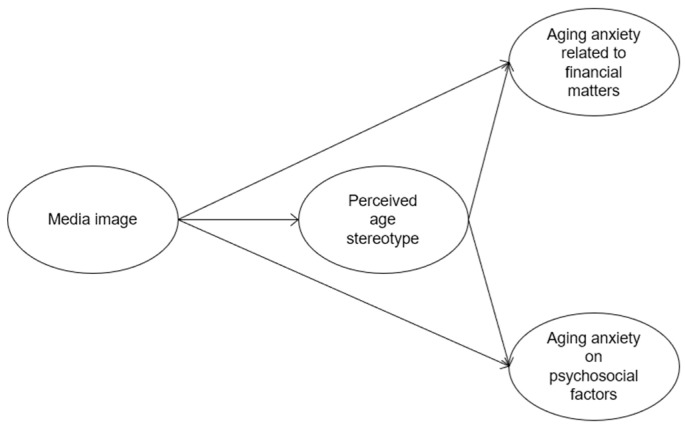
Conceptual model of the hypothesized relationships among media representation, perceived age stereotype, and aging anxiety.

The results of the structural model are illustrated in Figure 1, using the standardized path coefficients. The structural model exhibited a good fit for all of the model fit indices. Specifically, the indices yielded values of $\chi^{2}\left(88\right)$ = 193.300 (*p* < 0.001), CFI = 0.976, RMSEA = 0.045, and SRMR = 0.024.

In support of the first hypothesis, media representation had a significant effect on perceived age stereotypes (β = 0.380, *p* < 0.001). The second and third hypotheses were also supported, as media representation had a significant association with aging anxiety related to financial matters (β = 0.145, *p* < 0.01) and aging anxiety pertaining to psychosocial factors (β = 0.136, *p* < 0.01). However, the fourth hypothesis was rejected, and only the fifth hypothesis was supported regarding the relationship between perceived age stereotypes and aging anxiety pertaining to psychosocial factors (β = 0.205, *p* < 0.001).

**Figure 2:**
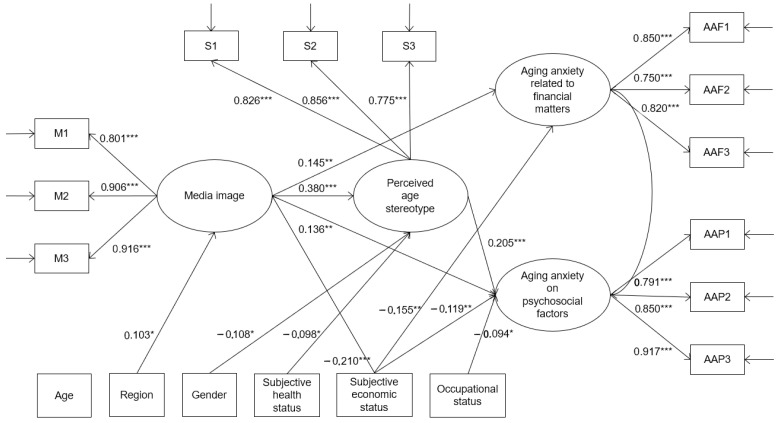
Structural equation model concerning the relationship between media image and aging anxiety related to financial matters and psychosocial factors with perceived age stereotype as a mediator. *Note*: M = media image; S = perceived age stereotype; AAF = aging anxiety related to financial matters; AAP = aging anxiety pertaining to psychosocial factors. Only significant paths are shown. Data are standardized coefficients. * *p* < 0.05, ** *p* < 0.01, and *** *p* < 0.001.

Regarding sociodemographic factors, region was significantly associated with media representation (β = 0.103, *p* < 0.05), while gender (β = −0.108, *p* < 0.05) and self-rated physical health status (β = −0.098, *p* < 0.05) were significantly associated with perceived age stereotypes. Subjective economic status was significantly associated with media representation (β = −0.210, *p* < 0.001), aging anxiety related to financial matters (β = −0.155, *p* < 0.01), and aging anxiety pertaining to psychosocial factors (β = −0.119, *p* < 0.01). Lastly, occupational status was significantly associated with aging anxiety pertaining to psychosocial factors (β = −0.094, *p* < 0.05). These findings indicate that the individuals living in metropolitan cities encountered more stereotypical representations of older adults in the media than those living in rural areas, and male participants as well as participants with greater perceived physical health statuses perceived themselves less stereotypically with respect to age. The participants who perceived their economic status to be good responded that they encounter fewer age-stereotypical representations in the media, and they exhibited less aging anxiety regarding both financial and psychosocial factors. Lastly, the employed participants displayed lower aging anxiety pertaining to psychosocial factors (see Figure 2).

### 3.4. Assessment of Mediation

The present study examines the potential mediating role of perceived age stereotypes in the relationship between media representation and aging anxiety. A bootstrapping analysis was conducted to assess the indirect effects at a 95% confidence level. The results, as presented in Table 3, reveal that only one pathway was found to be significant. Specifically, the indirect pathway from media representation to aging anxiety pertaining to psychosocial factors through the mediator of perceived age stereotype (95% CI = 0.034, 0.121) was significant, while the indirect pathway from media representation to aging anxiety related to financial matters was not significant (95% CI = −0.005, 0.081). These findings suggest that perceived age stereotypes may play a mediating role in the association between media representation and aging anxiety pertaining to psychosocial factors but not in the relationship between media representation and aging anxiety related to financial matters.

## 4. Discussion

This study examines the associations between media representations of aging, perceived age stereotypes, and aging anxiety among older Korean adults, with particular attention paid to the mediating role of perceived age stereotypes. The results indicate that greater exposure to negative portrayals of aging in the media is linked to stronger perceived age stereotypes and elevated levels of aging anxiety, including both psychosocial and financial dimensions. Importantly, perceived age stereotypes were found to mediate the relationship between media representations and psychosocial aging anxiety but not financial aging anxiety. These findings make several contributions to the existing literature and offer theoretical, cultural, and policy-relevant insights.

### 4.1. Theoretical Contributions

This study extends prior work on Stereotype Embodiment Theory (SET) by applying it to the domain of aging anxiety—a construct that reflects future-oriented psychological vulnerability. While the initial applications of SET primarily focused on physical health outcomes (Levy et al., 2000; Levy & Myers, 2004), recent research has increasingly explored its relevance to mental health, including depression, loneliness, and suicidal ideation (Gendron et al., 2024; Kang & Kim, 2022; H. Kim et al., 2019; Pedroso-Chaparro et al., 2023). These studies collectively underscore the broad psychological impact of internalized ageism, highlighting its relevance across diverse mental health outcomes. The current study builds on this growing trend by focusing on aging anxiety, an understudied yet critical psychological response to internalized age stereotypes. Moreover, while previous experimental work conducted by Levy et al. (2022) has demonstrated the short-term psychological effects of age-stereotypical messaging, our study investigates these processes in a real-world setting based on participants’ self-reported, cumulative exposure to ageist media content. This approach allows us to explore how routine encounters with age-related stereotypes in daily media may be linked to older adults’ internalized beliefs and their psychological well-being.

Our findings align most closely with SET’s psychological pathway, which describes how age stereotypes become internalized and influence self-perceptions and emotional well-being (Levy, 2009). SET posits that age stereotypes influence older individuals’ health through three primary pathways: physiological, behavioral, and psychological (Levy, 2009; Levy et al., 2000, 2021; Levy & Leifheit-Limson, 2009; Levy & Myers, 2004). The physiological pathway suggests that exposure to negative age stereotypes can trigger stress responses that affect biological systems. For instance, Levy et al. (2000) found that older adults exposed to negative age stereotypes exhibited significantly higher systolic and diastolic blood pressure and greater skin conductance, indicating that age-based stereotypes can act as a cardiovascular stressor. The behavioral pathway emphasizes the influence of age stereotypes on health-related behaviors. Older adults who internalize negative beliefs about aging may come to view health-promoting efforts as futile and thus reduce their engagement in behaviors like getting regular exercise, adhering to a diet with balanced nutrition, or attending preventive medical check-ups (Levy & Myers, 2004). Over time, this withdrawal from health-promoting behaviors can lead to worsened physical health and greater dependence. The psychological pathway—which is most closely aligned with the findings of the present study—focuses on the internalization of age stereotypes into one’s self-concept, leading to lower self-esteem, a reduced sense of control, and heightened anxiety or depressive symptoms. Levy and Leifheit-Limson (2009) demonstrated that self-perceptions of aging predicted memory decline, while Wurm et al. (2007) found that negative age-related cognition was linked to increased depressive symptoms and feelings of helplessness. In our study, perceived age stereotypes significantly mediated the relationship between media exposure and aging anxiety pertaining to psychosocial factors, suggesting that older adults may internalize negative media messages and apply them to their self-perceptions, thereby increasing anxiety about their cognitive and emotional functioning, social relevance, and psychological resilience.

This mediating role of perceived age stereotype is theoretically grounded in the psychological pathway of SET and supported by accumulating empirical evidence. For instance, Wurm et al. (2007) found that internalized negative age stereotypes were significantly associated with greater depressive symptoms and a reduced sense of control among older adults. Similarly, Shimizu et al. (2022) demonstrated that internalized age stereotypes predicted declines in cognitive performance and mental health over time, reinforcing the notion that self-directed ageist beliefs can translate into psychological vulnerabilities. These findings strengthen the interpretation that stereotype internalization acts as a meaningful intermediary process through which media-based ageism affects older adults’ psychological outcomes.

However, the absence of a mediating effect in the relationship between media exposure and financial aging anxiety highlights important boundary conditions of SET. While psychosocial concerns may be more shaped by stereotype-based influence, financial anxiety in older age likely stems from structural, economic, and policy-level realities, such as inadequate pensions, employment insecurity, or past financial hardship. In Korea, where nearly half of the older population lives in relative poverty (OECD, 2021), financial stress may be less reflective of self-perceptions and more reflective of material deprivation. This distinction underscores the need to conceptually separate domains of aging anxiety and extend SET by integrating economic and structural dimensions of aging. For instance, factors such as pension adequacy, financial literacy, and economic hardship have been linked to older adults’ financial well-being and anxiety (Kadoya et al., 2018).

### 4.2. Practical Implications

These findings have practical implications. Media, as a powerful shaper of social norms, can play a critical role in either reinforcing or dismantling ageist beliefs. To mitigate aging anxiety, especially in the psychosocial domain, there is a pressing need for age-positive representations in mass media. Highlighting the diversity, capability, and social contributions of older adults can counterbalance prevalent narratives of frailty and decline (Chakraborty et al., 2021). Additionally, educational interventions that promote intergenerational understanding from an early age may foster more resilient and stereotype-resistant identities.

The significant association between media portrayals and aging anxiety calls for the development of age-inclusive media content guidelines that actively discourage the dissemination of ageist narratives. To counteract ageist narratives, Korean media stakeholders should take initiative in promoting inclusive portrayals of older adults, highlighting their strengths, capabilities, and diverse contributions beyond stereotypes of dependency. Such efforts are crucial not only for reducing aging anxiety but also for reshaping societal attitudes toward aging in a rapidly aging society. These efforts will play a vital role in both reducing aging anxiety and cultivating more age-positive social norms in rapidly aging societies like Korea.

### 4.3. Limitations

While the results of this study provide valuable insights into reducing aging anxiety among older adults in today’s aging society, this study also has some limitations. One limitation is the use of cross-sectional data, which only allows for the description of associations between major variables at a single point in time, which limits the ability to draw causal inferences between media exposure, stereotype internalization, and aging anxiety. While the associations identified are consistent with theoretical pathways proposed by SET, future research employing longitudinal designs is necessary to clarify the directionality and causal nature of these relationships.

Moreover, the current study can be improved by investigating the effects of positive representations of aging in the media on age stereotypes and aging anxiety among older adults. While SET suggests that positive media representations of aging can buffer individual stress and promote positive health behaviors, ultimately leading to beneficial health effects on older individuals, the current study only focused on the negative effects of media portraying aging negatively. With more efforts being made to display diverse and positive representations of older adults and the aging process in the media, investigating the relationship between positive media representations and other associated variables, such as self-esteem or perceived social support, could also facilitate further understanding of the mechanisms behind SET and its impact on the mental health of older adults.

## 5. Conclusions

This study has significant implications for understanding aging anxiety among older adults in the context of population ageing. First, this study contributes to previous bodies of literature on aging anxiety by examining both the financial and psychosocial dimensions of this phenomenon. As aging anxiety has been found to have negative associations with older adults’ health, including increased depressive symptoms, loneliness, apprehension regarding future prospects, disability, and cognitive impairment, it is crucial to address this issue in order to promote healthier and happier later lives for older adults (Bergman & Segel-Karpas, 2021). This is not only important from an individual perspective but also from a societal standpoint, as decreased productivity and increased healthcare expenditure can have economic consequences for a nation. Second, this study is particularly meaningful as it investigates the role of media in relation to aging anxiety. Despite the continuous expansion of the media’s influence on shaping people’s perceptions of old age and aging, few studies have explored this relationship. Given that many aspects of our lives are becoming increasingly contact-free and online-based, it would now be prudent to investigate the impact of media on our psychological health. Third, the findings of this study highlight the role of perceived age stereotypes in the relationship between media and aging anxiety within the framework of SET. Based on the pathways that link media exposure to aging anxiety, practitioners can identify which specific elements to focus on when attempting to reduce levels of aging anxiety among older adults. Overall, this study contributes to our understanding of aging anxiety and its potential causes as well as the role of media in shaping perceptions of aging. By addressing aging anxiety among older adults, we can promote healthy aging and improve older individuals’ quality of life while also benefiting society as a whole.

## Figures and Tables

**Table 1:** Sociodemographic characteristics of the study participants (*N* = 600).

Characteristics	n (%)	Range
Age (M, SD)	73.77 (5.797)	65–84
Gender		
Male (=1)	266 (44.3)	
Female (=0)	334 (55.7)	
Region		
Rural city (=1)	256 (21.3)	
Medium-sized city (=2)	415 (34.6)	
Metropolitan city (=3)	529 (44.1)	
Spouse		
With spouse (=1)	374 (62.3)	
Without spouse (=0)	226 (37.7)	
Housing status		
Participant owns a house (=1)	461 (76.8)	
Participant does not own a house (=0)	139 (23.2)	
Occupational Status		
Employed (=1)	243 (40.5)	
Not employed (=0)	357 (59.5)	
Subjective health status (M, SD)	3.09 (0.908)	1–5
Subjective economic status (M, SD)	2.40 (0.681)	1–5

**Table 2:** Factor loadings for the measurement model.

Measured Variable	Unstandardized Factor Loading	SE	Standardized Factor Loading
Media image			
M1	1.000	─	0.802
M2	1.043	0.041	0.906
M3	1.076	0.042	0.915
Perceived age stereotypes			
S1	1.000	─	0.826
S2	1.146	0.054	0.859
S3	1.030	0.053	0.772
Aging anxiety: financial			
AAF1	1.000	─	0.850
AAF2	1.001	0.052	0.750
AAF3	1.017	0.048	0.820
Aging anxiety: psychosocial			
AAP1	1.000	─	0.791
AAP2	1.020	0.045	0.850
AAP3	1.126	0.046	0.917
$\chi^{2}$ (48) = 126.361, *p* = 0.000, CFI = 0.982, RMSEA = 0.052, SRMR = 0.0246			

*Note*. *N* = 600. All factor loadings are significant at the 0.001 level. M = media image; S = perceived age stereotype; AAF = aging anxiety related to financial matters; AAP = aging anxiety pertaining to psychosocial factors.

**Table 3:** Results of mediation effect.

	95% Confidence Interval
Media image → perceived age stereotypes → aging anxiety: financial	[−0.005, 0.081]
Media image → perceived age stereotypes → aging anxiety: psychosocial	[0.034, 0.121]

## Data Availability

The data presented in this study are only available on request from the corresponding author due to privacy and ethical constraints.
